# Supplementary material for: Natural variation of TBR confers plant zinc toxicity tolerance through root cell wall pectin methylesterification
Source: Nat Commun. 2024 Jul 11;15:5823. doi: 10.1038/s41467-024-50106-5 (PMC11239920; doi:10.1038/s41467-024-50106-5)
Supplement: Supplementary file 1 — Supplementary Information [file 41467_2024_50106_MOESM1_ESM.pdf]

Natural variation of *TBR* confers plant zinc toxicity tolerance through root cell wall pectin methylesterification

Zhong *et al*

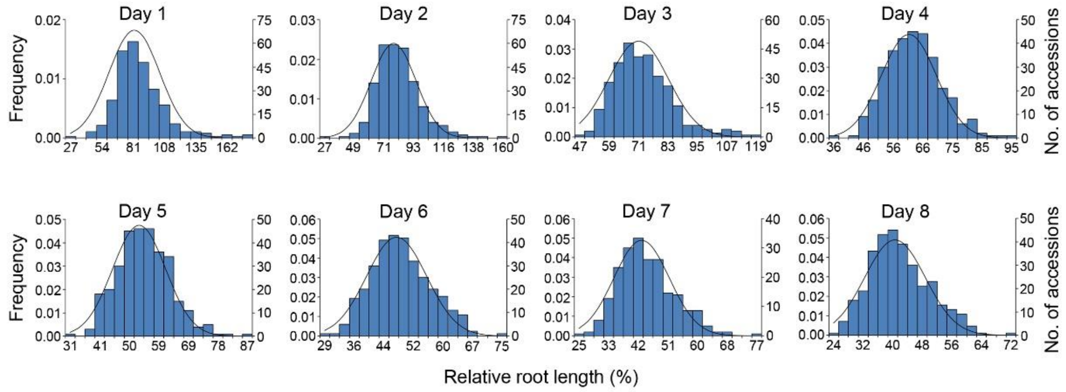

Supplementary Figure 1. Distribution of relative primary root length in high Zn conditions of 317 accessions. Histograms for the relative primary root length in high Zn conditions (root length in high Zn / root length in control) over a 8-day time course. x-axis: relative primary root length; y-axis: frequency (left) and the number of accessions (right).

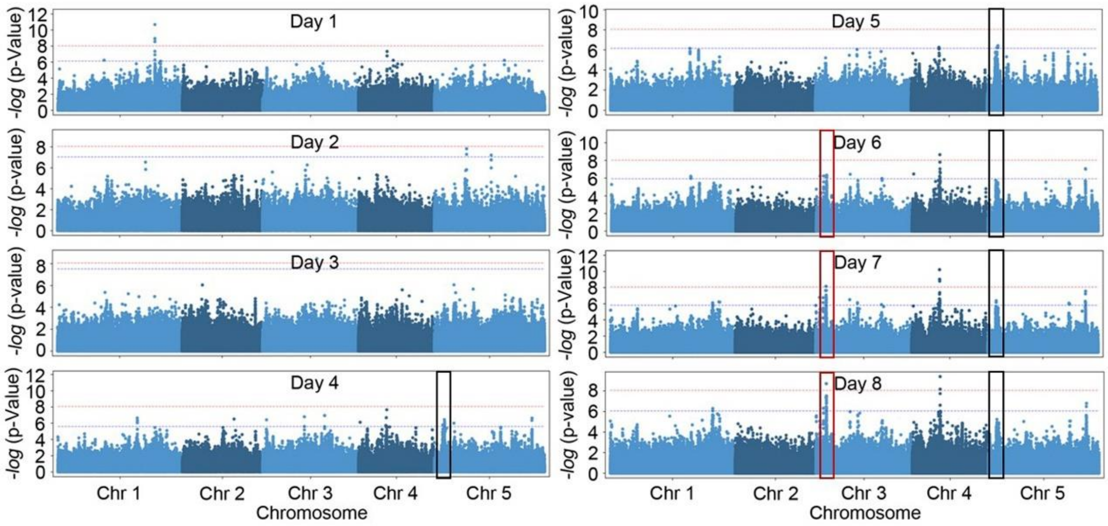

Supplementary Figure 2. GWAS for the relative primary root length in high Zn conditions in *Arabidopsis.* Manhattan plots of GWAS analysis using the relative primary root length (root length in high Zn / root length in control) in high Zn conditions at each day of 8-day time courses after germination. x-axis: SNP position. y-axis: -$\log_{10}$ p-value of association of the SNPs according to AMM mixed model analysis. Chromosomes are depicted in different colors. The horizontal blue dash line corresponds to a 5% significance threshold after Benjamini Hochberg correction and the red dashed line corresponds to a 5% significance threshold after Bonferroni correction. The red boxes indicate the peak around the *FRD3* locus and the black boxes indicate the peak surrounding the *TBR* locus.

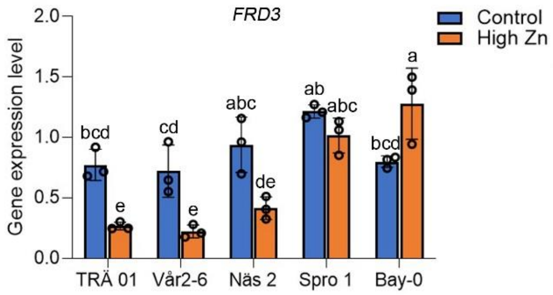

Supplementary Figure 3. Gene expression level of *FRD3* in roots of other extreme accessions. (Sensitive accession: TRÄ 01, Vår2-6 and Näs 2; Tolerant accessions: Spro 1 and Bay-0). Expression levels were normalized to expression of TRÄ 01 in control conditions. Data are mean ± S.D. Circles indicate a single biological replication, n=3. Statistical analysis was performed using one-way ANOVA analysis with Tukey's HSD test ($p < 0.05$). Source data are provided as a Source Data file.

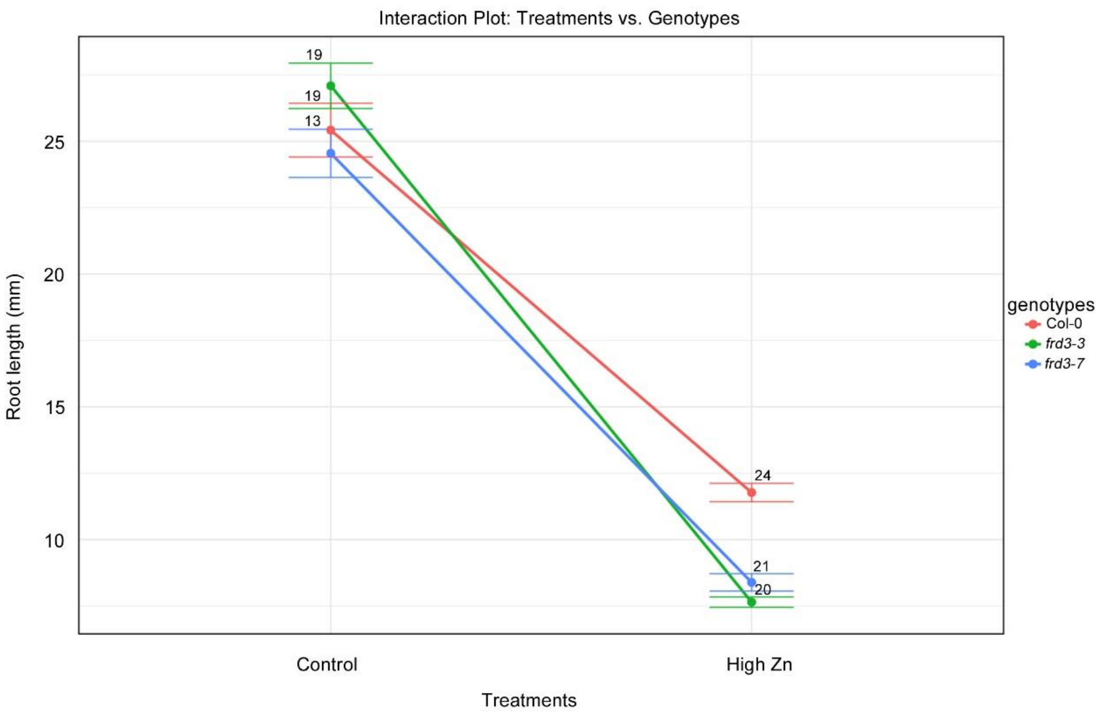

Supplementary Figure 4. Interaction plot of treatments and genotypes between *frd3* mutants and Col-0 wildtype. Root length (y-axis) in control medium and high Zn medium. Data are mean ±S.E.M. The number on each error bar indicates biologically independent samples. Statistical analysis was performed using two-way ANOVA analysis with Tukey's HSD test ($p < 0.05$).

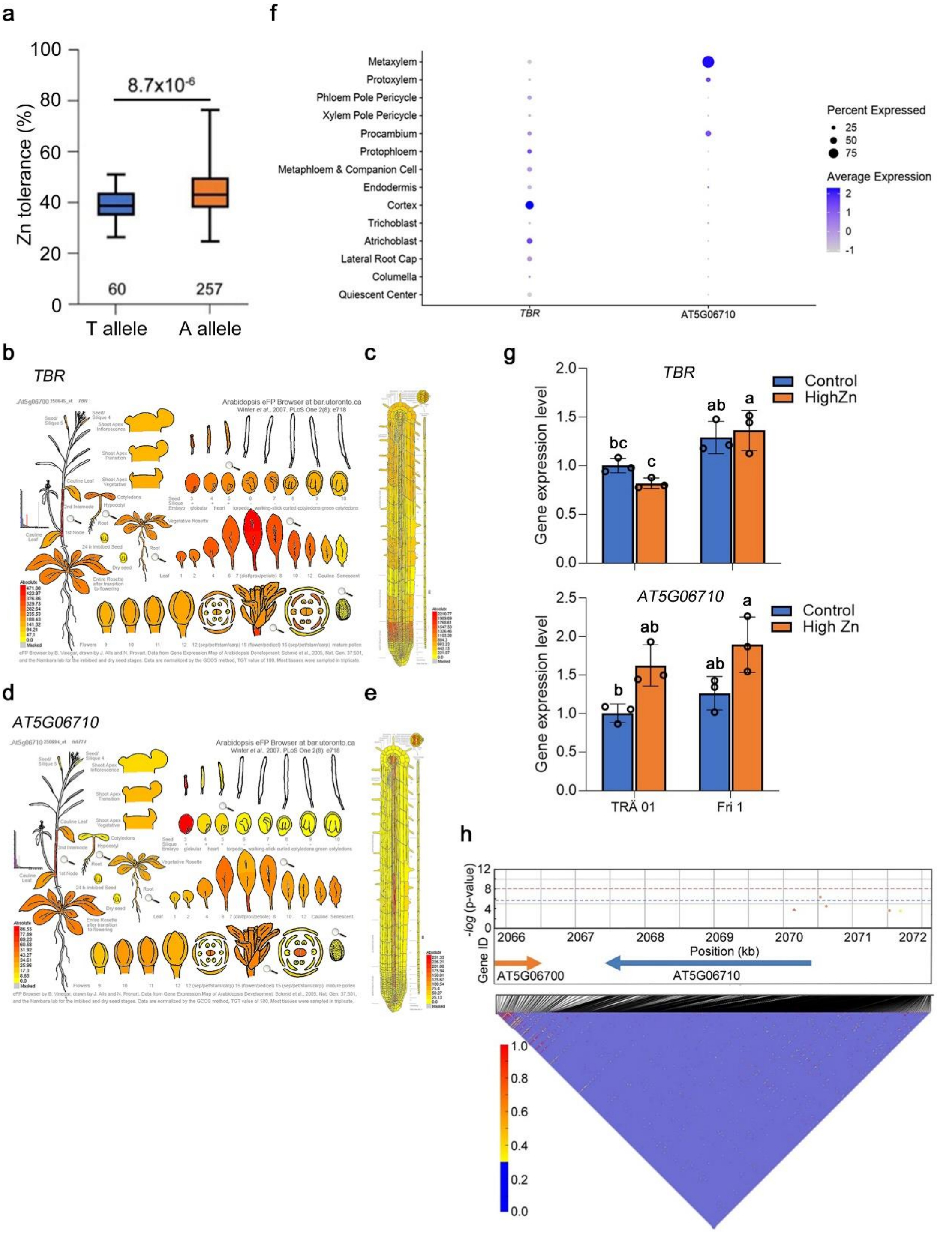

Supplementary Figure 5. Analysis of the top SNP by GWAS and gene expression patterns of candidate genes. a Boxplots for Zn tolerance of two variants grouped by the top SNP at 7 DAG. The number corresponds to p-value analyzed by two-tailed Student's *t*-test. The number below each box indicates the number of accessions, the horizontal black lines indicate the median and the two whiskers represent the interquartile range. **b, c** Expression pattern of *TBR* from publicly available microarray datasets in various tissues (**b**) and in roots (**c**). Figure was obtained from the Arabidopsis eFP browser (https://bar.utoronto.ca/efp/cgi-bin/efpWeb.cgi) under a CC-BY 4.0 licence (https://creativecommons.org/licenses/by/4.0/) and was not modified. **d, e** Expression pattern of

AT5G06710 from microarray datasets in various tissues (**d**) and in roots (**e**). Figure was obtained from the Arabidopsis eFP browser (https://bar.utoronto.ca/efp/cgi-bin/efpWeb.cgi) under a CC-BY 4.0 licence (https://creativecommons.org/licenses/by/4.0/) and was not modified. **f** Single cell atlas-based cell-type expression pattern in roots of *TBR* and *AT5G06710* based on data from Shahan et al. 2022 and visualized with the dotplot feature of Seurat in R. Dot size represents the percentage of cells in which each gene is expressed (% expressed). Dot colors indicate the average scaled expression of each gene in each cell-type group with solid colors indicating higher expression levels. **g** Transcript levels of *TBR* and *AT5G06710* in roots of accessions with contrasting root responses to high Zn (Sensitive accession: TRÄ 01; Tolerant accession: Fri 1). Expression levels were normalized to expression of TRÄ 01 in control conditions. Data are mean $\pm$ S.D. Circles indicate a single biological replication, n=3. Statistical analysis was performed using one-way ANOVA analysis with Tukey's HSD test ($p < 0.05$). **h** Linkage disequilibrium (LD) plot for the top SNP (2,070,693) by GWAS in Zn tolerance. -log(p-value) of associated SNPs shown on y-axis; the candidate region, SNPs position and candidate genes on chromosome 5 shown on x-axis. Source data are provided as a Source Data file.

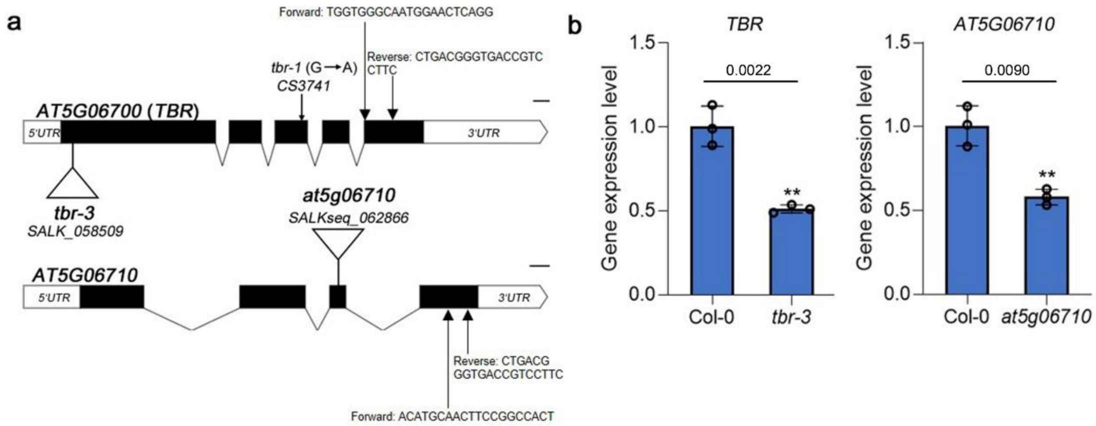

Supplementary Figure 6. Genomic structures of candidate genes and gene expression level of the *tbr-3* mutants and *at5g06710*. a The genomic structures of *TBR* and *AT5G06710* and the T-DNA insertion sites or mutation sites. Black boxes indicate exons. Scale bar: 100 bp. Gene models were generated by Exon-intron graphic maker (http://wormweb.org/exonintron). The primers on the genomic structure were used for transcript analysis in **b**. **b** Transcript analysis of *TBR* in *tbr-3* mutants and of *AT5G06710* in *at5g06710* mutant. Expression levels were normalized to expression of Col-0 in control conditions. Data are mean ± S.D. Circles indicate a single biological replication, n=3. The number corresponds to p-value analyzed by two-tailed Student's *t*-test. Source data are provided as a Source Data file.

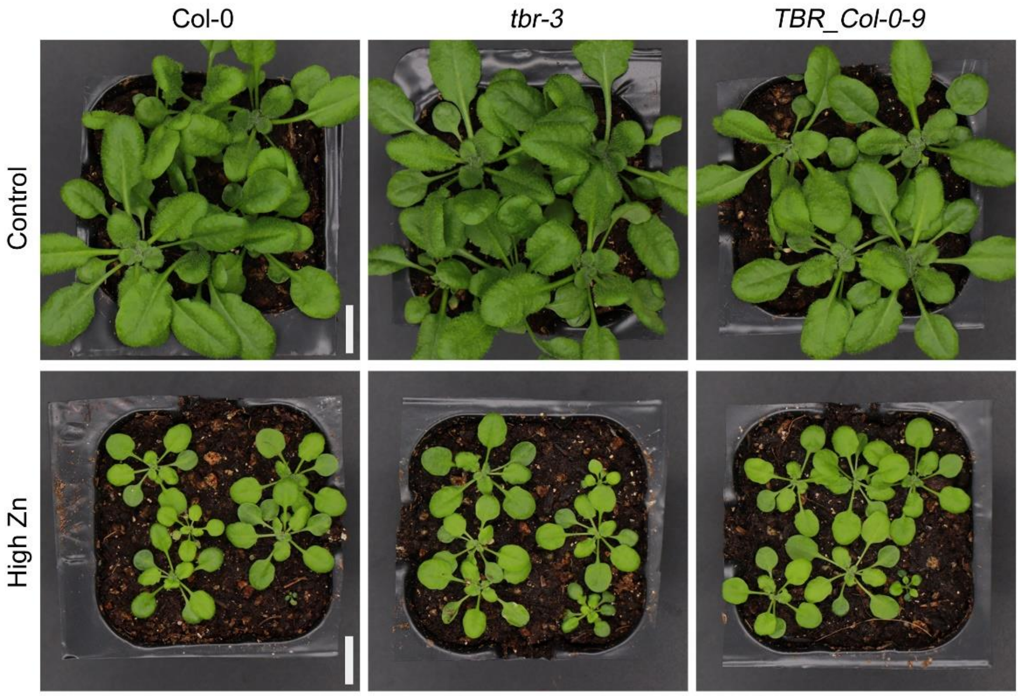

Supplementary Figure 7. Plant vigor of wildtype, *tbr*-3 mutant and *TBR_Col-0-9* complemented line in high Zn soils. Seedlings of wildtype, *tbr*-3 mutant, and *TBR_Col-0-9* grown under control and high Zn (600 mg/kg) soils for 4 weeks after transferring to the soil. Scale bars: 10 mm.

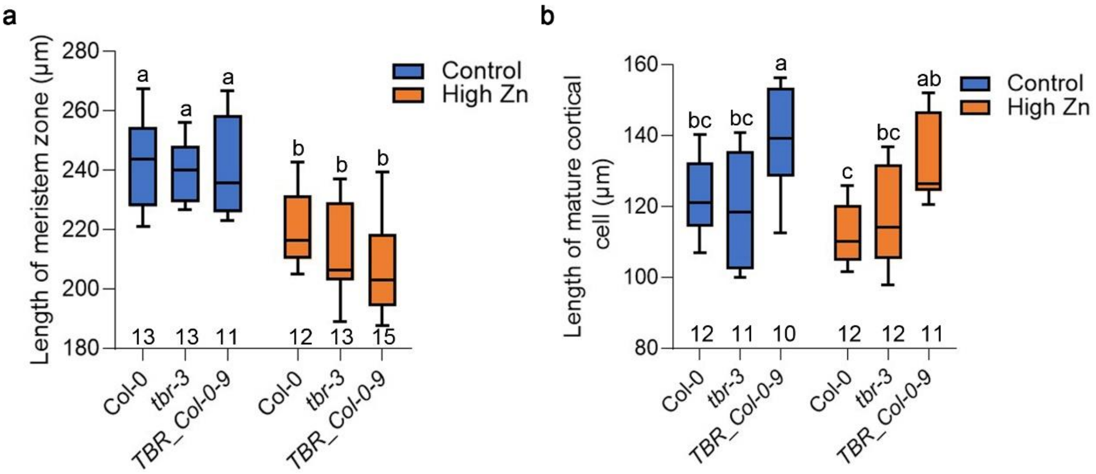

Supplementary Figure 8. Root traits at the cellular level of wildtype, *tbr-3* and *TBR_Col-0-9*. **a, b** Boxplots for meristem zone length (**a**) and mature cortical cell length (**b**) under control and high Zn (300 μM) medium at 4 DAG, respectively. The number below each box indicates the number of replicates. Statistical analysis was performed using one-way ANOVA analysis with Tukey's HSD test ($p < 0.05$). For box plots, the horizontal line represents the median value, the lower and upper quartile represent 25th and 75th percentile and whiskers show the maximum and minimum values. Source data are provided as a Source Data file.

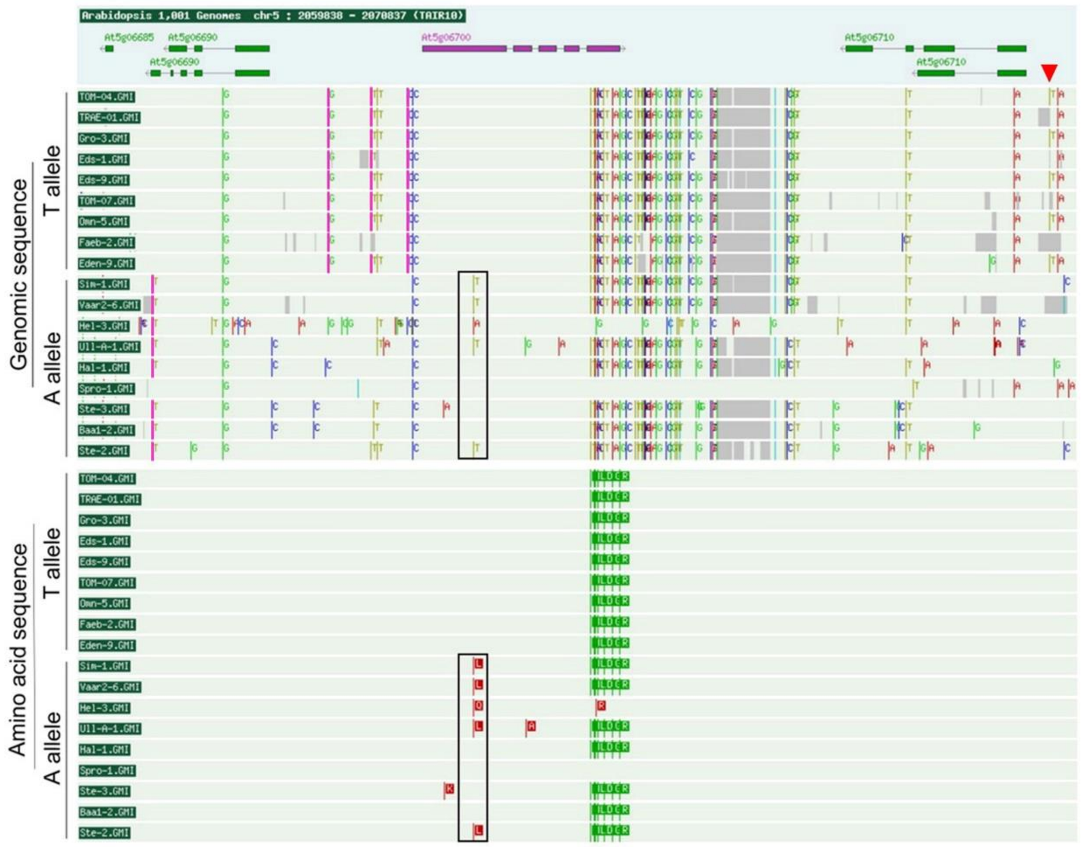

Supplementary Figure 9. Polymorphism analysis around the *TBR* locus in representative accessions. Comparison of SNPs and amino acid sequences of *TBR* in representative accessions of T and A allelic groups (with respect to the *TBR* lead SNP that is highlighted with a red triangle). Data are from the 1001 *Arabidopsis* genomes project (http://signal.salk.edu/atg1001/3.0/gebrowser.php). SNPs or amino acid substitutions related to the reference sequence of Col-0 are indicated by letters. Black boxes indicate non-synonymous SNP and amino acid substitution which is in A-allelic group accessions. Magenta lines represent SNPs variation in the promoter region and 5'-UTR.

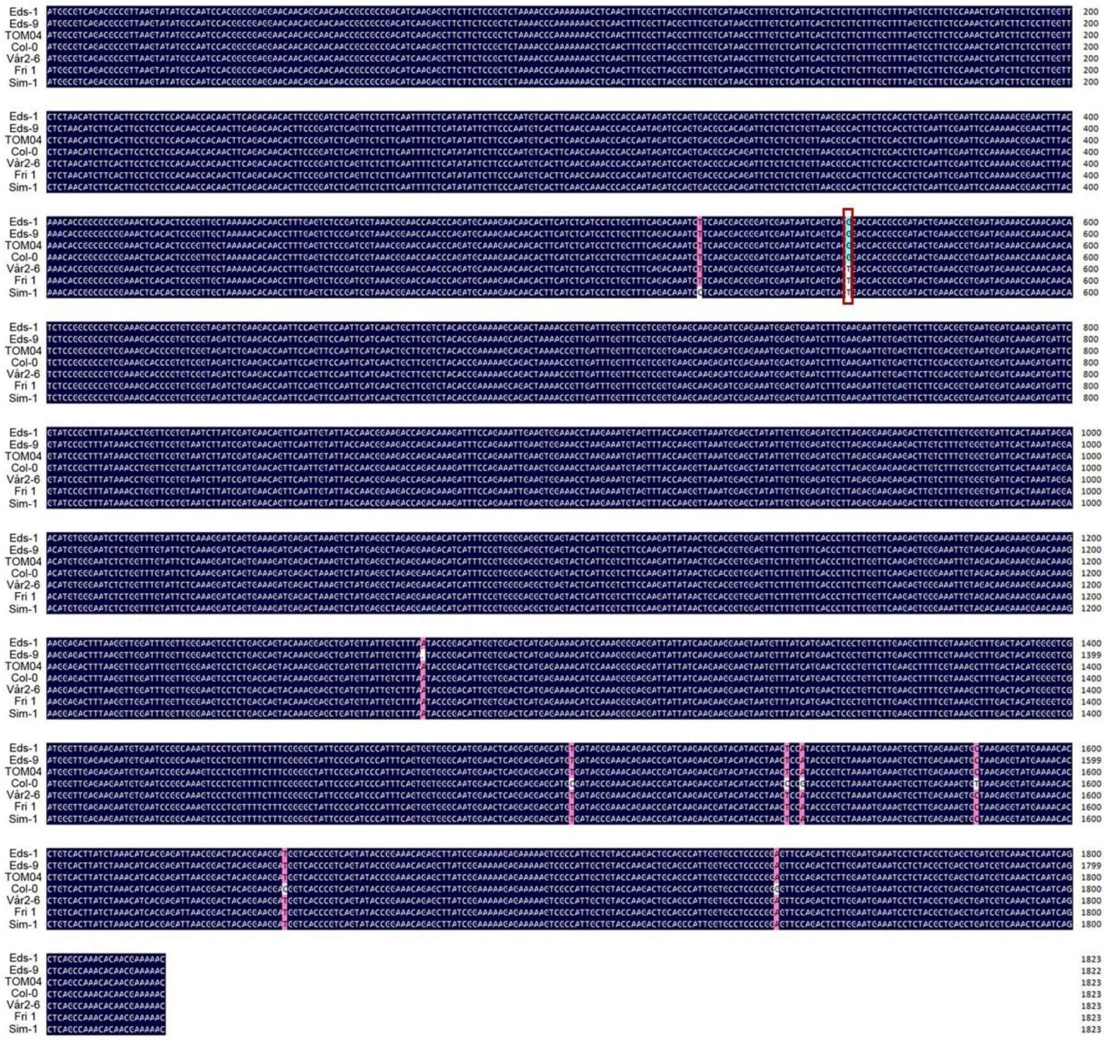

Supplementary Figure 10. Comparison of *TBR* coding sequence (from the translational start codon to stop codon, 1824 bp) between T-allelic accessions (Eds-1, Eds-9 and TOM 04) and A-allelic accessions (Col-0, Vår2-6, Fri 1 and Sim-1). Sequence variants are indicated in different colors. Red box indicates the SNP variation at position 2,070,324.

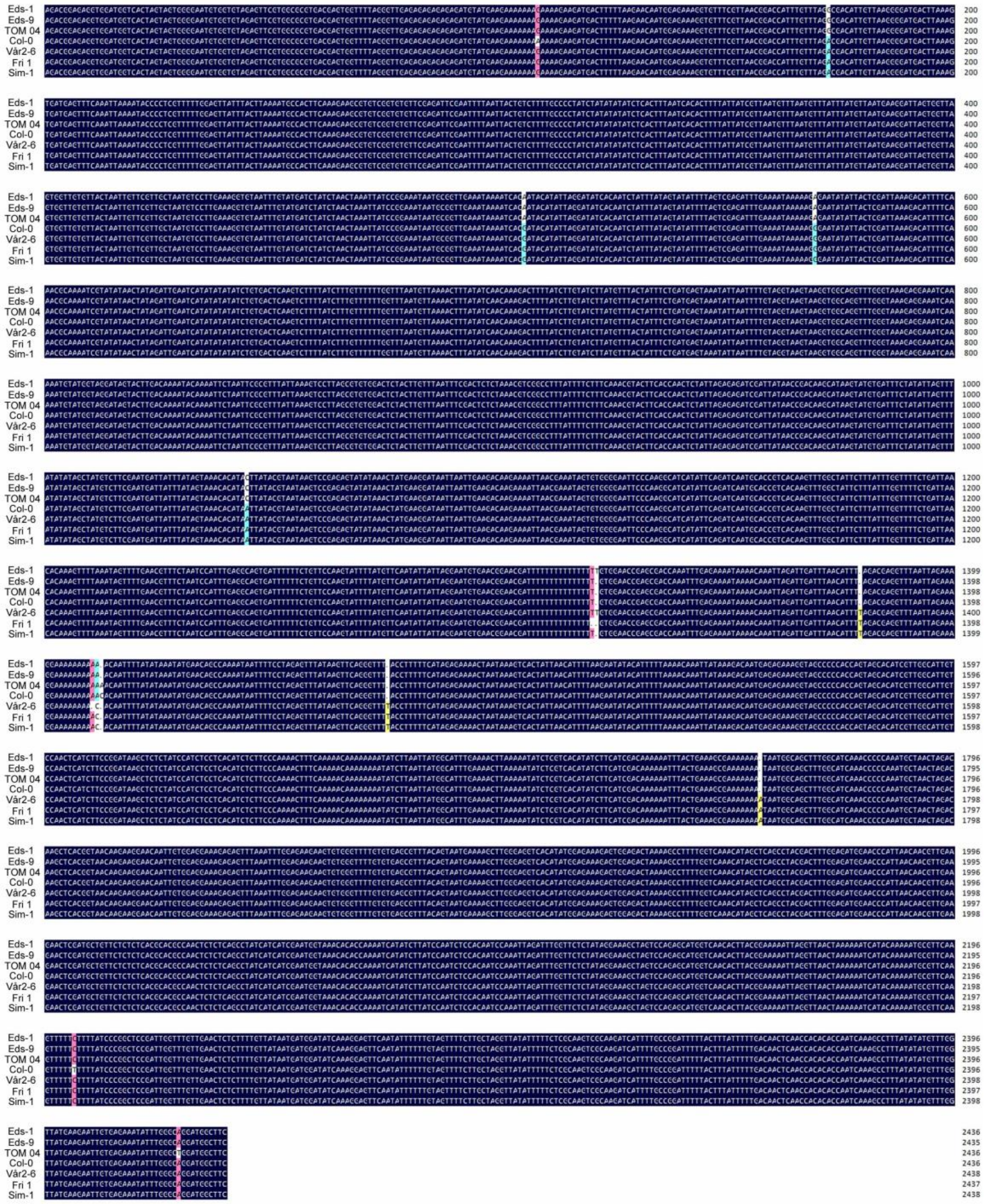

Supplementary Figure 11. Comparison of *TBR* promoter sequences and 5'-UTR between T-allelic accessions (Eds-1, Eds-9 and TOM 04) and A-allelic accessions (Col-0, Vår2-6, Fri 1 and Sim-1). Sequence variants are indicated in different colors.

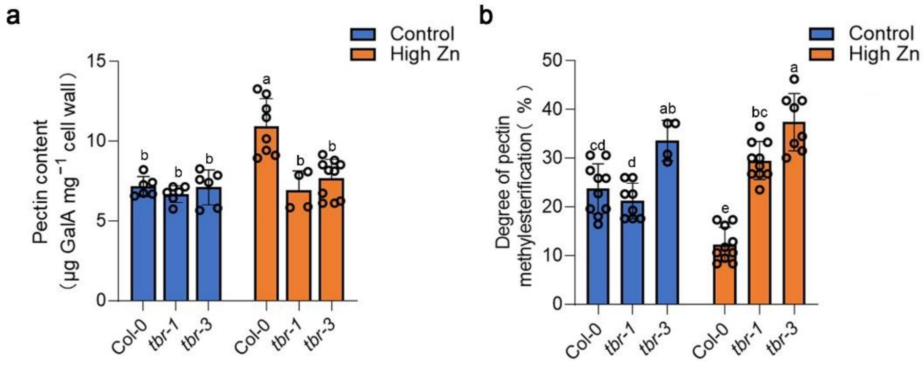

Supplementary Figure 12. *TBR* influences pectin methylesterification in root cell walls. a, b Pectin content (**a**), and pectin methylesterification degree (**b**) in roots of wildtype, *tbr-3* and *TBR_Col-0-9* grown in control and high Zn (150 μM) medium for 14 DAG. Circles indicate a single biological replication with two technical replicates, *n*=6, 6, 6, 8, 4, 10 in **a;** *n*=10, 8, 4, 10, 10, 8 in **b**. Data are mean ± S.D. Statistical analysis was performed using one-way ANOVA analysis with Tukey's HSD test ($p < 0.05$). Source data are provided as a Source Data file.

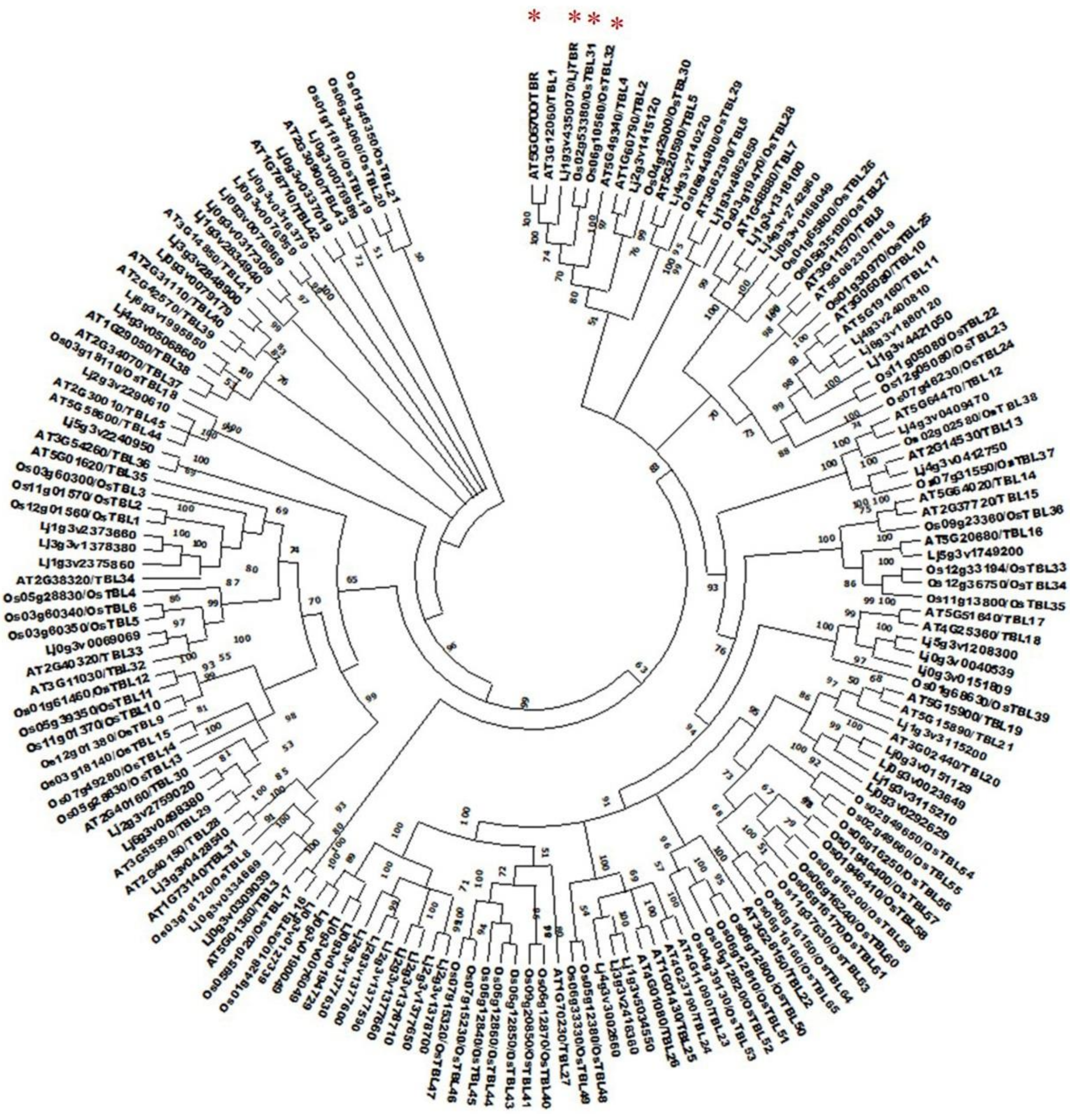

Supplementary Figure 13. Phylogenetic analysis of TBL family *from Arabidopsis thaliana*, *Oryza sativa* and *Lotus japonicus*. MEGA 10 was used to construct a neighbor-joining phylogeny (bootstrap replications = 1,000). Asterisks represent TBR, LjTBR, OsTBL31 and OsTBL32.

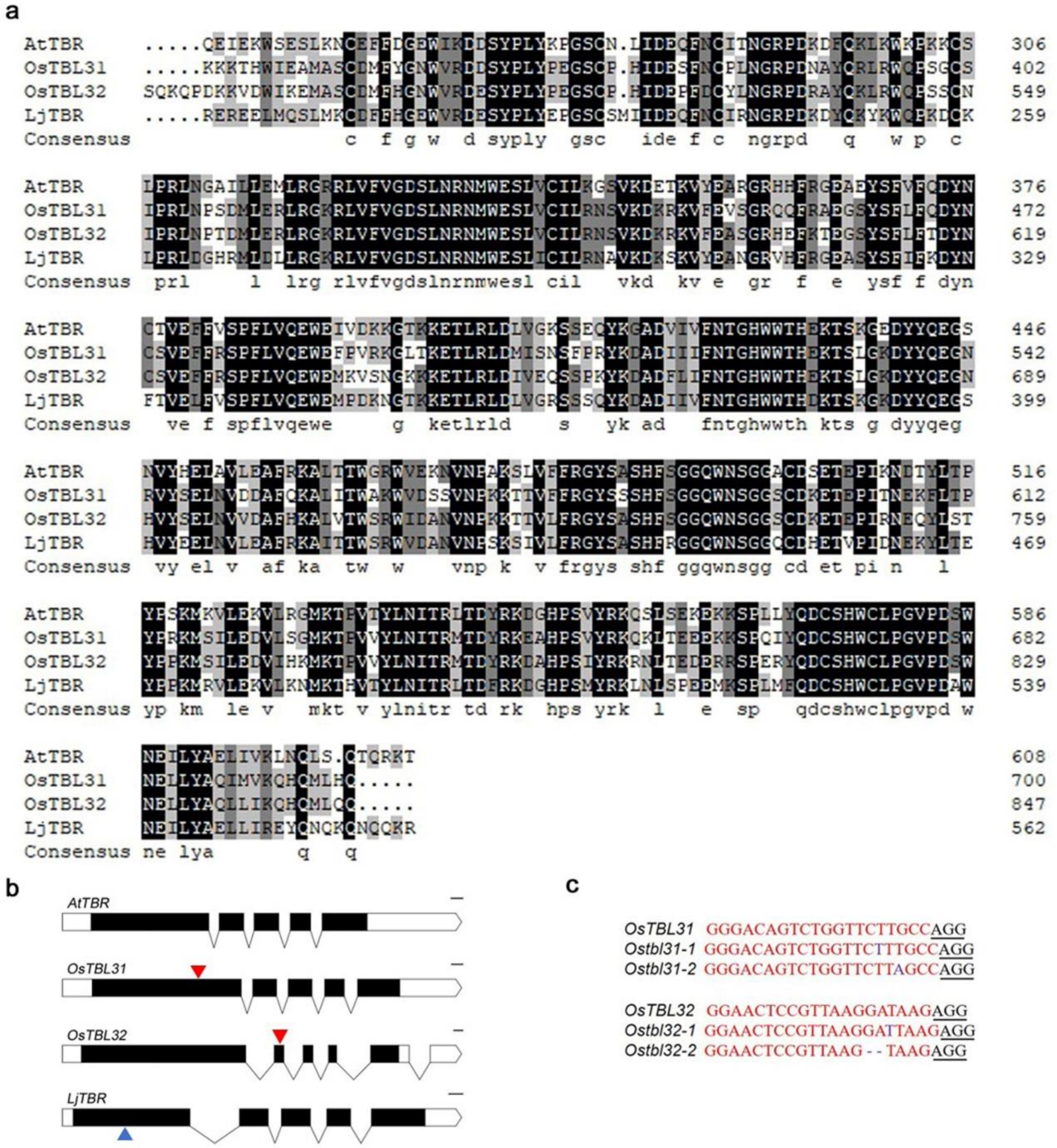

Supplementary Figure 14. Alignment and gene structure of *TBR* in different plant species. a Partial amino acid sequence alignment of TBR in *Arabidopsis thaliana*, *Oryza sativa* and *Lotus japonicus*. **b** Gene structure of *AtTBR*, *OsTBL31*, *OsTBL32* and *LjTBR*. Scale bar, 100 bp. Red triangles represent the target position for guide RNAs in gene edited lines; bule triangle represents insertion position of LORE1 retrotransposon. **c** Mutations in *OsTBL31* and *OsTBL32* by sequencing the target site in T_0 transgenic lines. The letters on the black line indicate the protospacer adjacent motif (PAM). Purple letters indicate the mutation pattern.

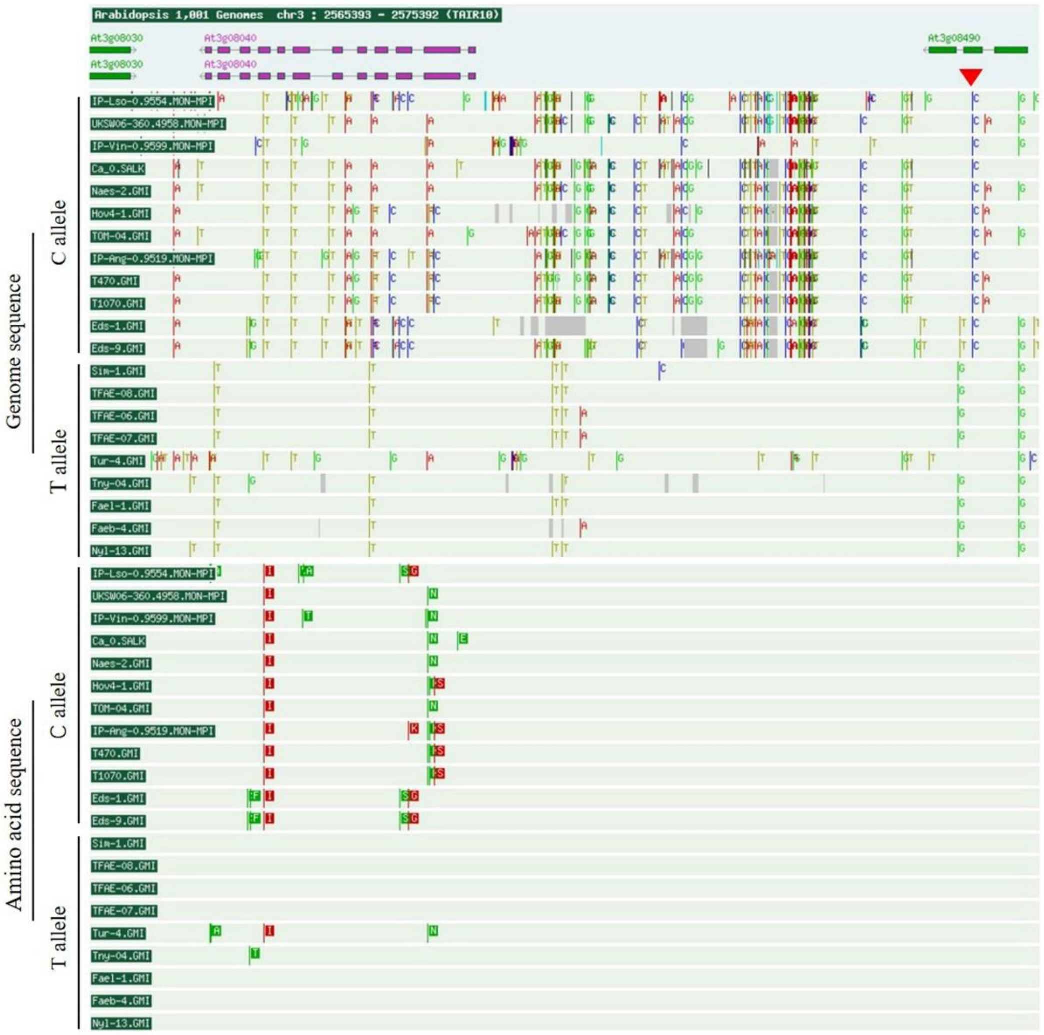

Supplementary Figure 15. Polymorphisms around the *FRD3* locus in representative accessions. Comparison of SNPs and amino acid sequences of *FRD3* in representative accessions of C and T allelic groups (with respect to the *FRD3* lead SNP that is highlighted with a red triangle). Data are from the 1001 *Arabidopsis* genomes site (http://signal.salk.edu/atg1001/3.0/gebrowser.php). SNPs and amino acid substitution related to reference sequence of Col-0 are indicated by letters.

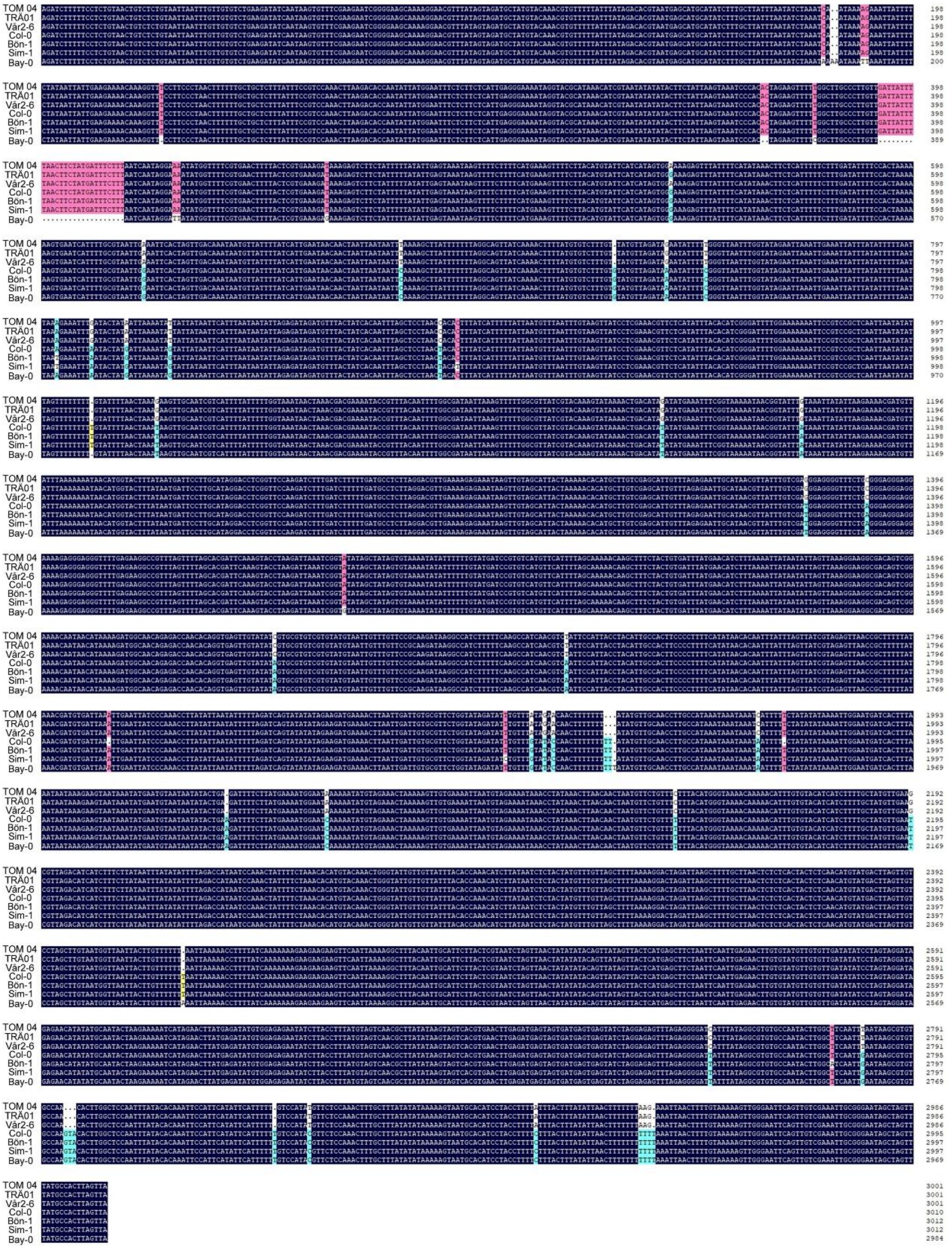

Supplementary Figure 16. ***FRD3*** **promoter sequences in extreme accessions** (TOM 04, TRÄ01 and Vår2-6 are C-allelic accessions, and Col-0, Bön-1, Sim-1 and Bay-0 are T-allelic accessions). Sequence variants are indicated in different colors.

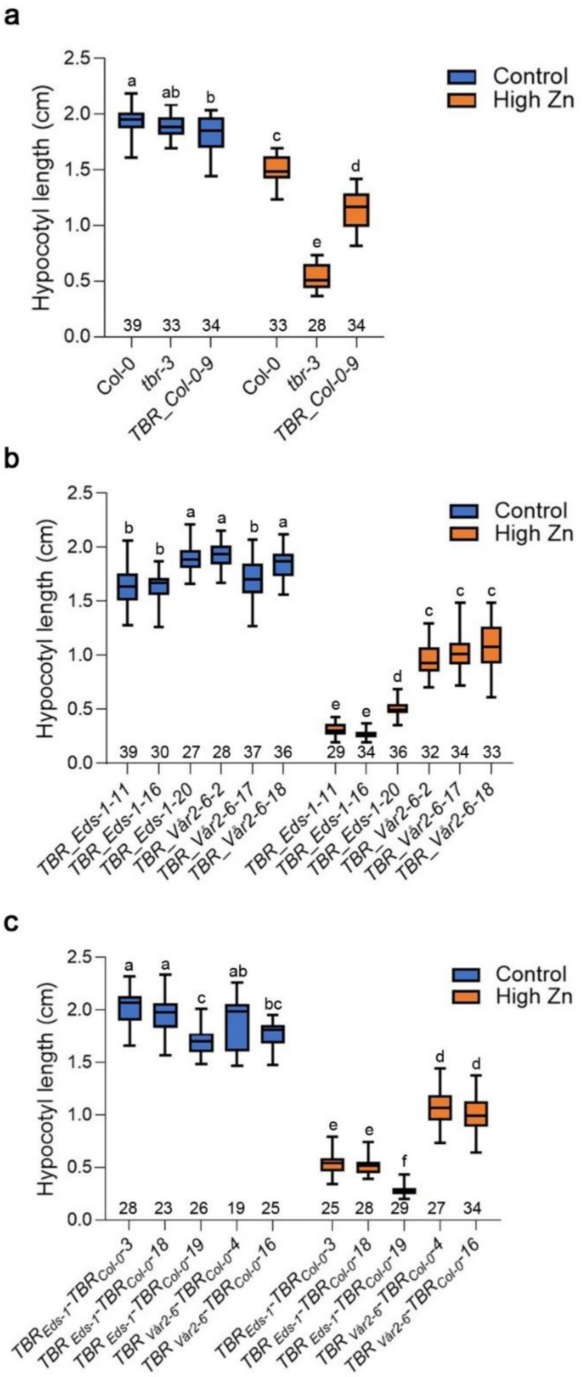

Supplementary Figure 17. *TBR* influences hypocotyl growth in high Zn. Box plots for hypocotyl length of Col-0, *tbr-3* and *TBR_Col-0-9* (**a**), T3 transgenic lines from *TBR_Eds-1* and *TBR_Vår2-6* (**b**), and T3 transgenic lines from $TBR_{Eds\text{-}1}$-$TBR_{Col\text{-}0}$ and $TBR_{\text{Vår2-6}}$-$TBR_{Col\text{-}0}$ (**c**) in control and high Zn (300 μM) medium in continuous darkness for 4 DAG. The T3 transgenic lines are single, homozygous insertions. The number below each box indicates the number of replicates. Statistical analysis was performed using one-way ANOVA analysis with Tukey's HSD test ($p < 0.05$). For box plots, the horizontal line represents the median value, the lower and upper quartile represent 25th and 75th percentile and whiskers show the maximum and minimum values. Source data are provided as a Source Data file.

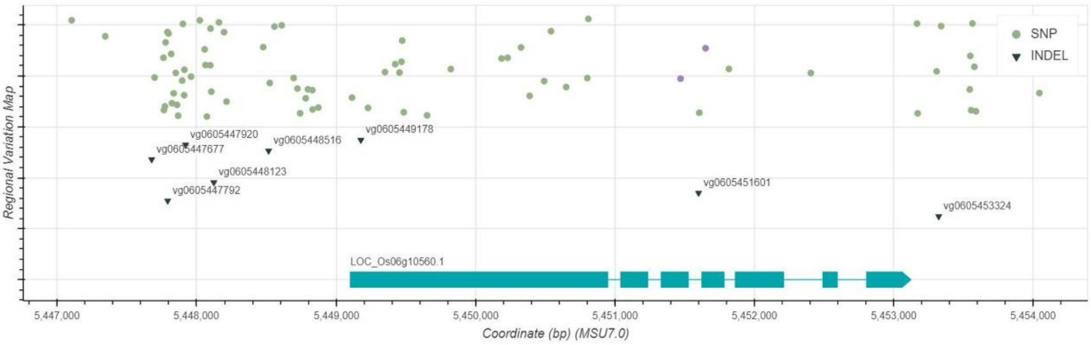

Supplementary Figure 18. Genomic variation of *OsTBL32* in rice. 86 sequence variants including 78 SNPs and 8 INDELs were found between 2.0 kb upstream and 1.0 kb downstream of *OsTBL32* from 4729 accessions using RiceVarMap 2.0.

Supplementary Table 1. Broad sense heritability of primary root length of 317 natural accessions.

	Day1	Day 2	Day 3	Day 4	Day 5	Day 6	Day 7	Day 8
**Control**	0.364	0.398	0.431	0.427	0.454	0.508	0.583	0.607
**High Zn**	0.470	0.512	0.507	0.525	0.577	0.650	0.671	0.701

Supplementary Table 2. Two-way ANOVA genotype-treatment interaction of root length in *frd3-3* mutant.

	Df	Sum	Mean	F	Pr(>F)	
**Genotypes**	1	30.637	30.637	3.506	0.065	
**Treatments**	1	5558.974	5558.974	636.100	0.000	***
**Genotypes:Treatments**	1	170.541	170.541	19.515	0.000	***

Significance codes: 0, ‘***’; 0.001, ‘**’; 0.01, ‘*’; 0.05, ‘.’; 0.1, ‘,’

Supplementary Table 3. Two-way ANOVA genotype-treatment interaction of root length in *frd3-7* mutant.

	Df	Sum	Mean	F	Pr(>F)	
**Genotypes**	1	82.757	82.757	10.223	0.002	***
**Treatments**	1	4058.057	4058.057	501.317	0.000	***
**Genotypes:Treatments**	1	28.766	28.766	3.554	0.063	

Significance codes: 0, '***'; 0.001, '**'; 0.01, '*'; 0.05, '.'; 0.1, ','

Supplementary Table 4. Quantification of western blot signals of *TBR* allelic transgenic lines.

Transgenic line	Control			High Zn		
	Intensity of TBR signal	Intensity of actin signal	Intensity of ratio	Intensity of TBR signal	Intensity of actin signal	Intensity of ratio
TBR_Eds-1-11	10018.71	47455.10	0.21	2774.32	43189.46	0.06
TBR_Eds-1-16	9709.10	45051.00	0.22	3124.30	40541.92	0.08
TBR_Vår2-6-17	43673.70	48139.63	0.91	52675.58	55429.39	0.95
TBR_Vår2-6-18	48517.75	48035.22	1.01	49731.56	56039.15	0.89

Supplementary Table 5. Transcription factor binding motifs in the promoter of *TBR* gene from Cistrome database.

Chromosome	Binding sites		Dap_motif gene
	Start	End	
**Chr5**	2063050	2063061	AtHB32

Supplementary Table 6. Quantification of western blot signals of *TBR* promoter allelic transgenic lines.

Transgenic line	Control			High Zn		
	**Intensity of TBR signal**	**Intensity of actin signal**	**Intensity of ratio**	**Intensity of TBR signal**	**Intensity of actin signal**	**Intensity of ratio**
$TBR_{Eds\text{-}1}$-$TBR_{Col\text{-}0}$-18	4677.45	18130.86	0.26	2409.69	14739.33	0.16
$TBR_{Eds\text{-}1}$-$TBR_{Col\text{-}0}$-19	3978.23	15915.91	0.25	1334.45	19293.28	0.07
$TBR_{Vår2\text{-}6}$-$TBR_{Col\text{-}0}$-4	10875.15	15657.45	0.69	8261.74	20263.45	0.41
$TBR_{Vår2\text{-}6}$-$TBR_{Col\text{-}0}$-16	18834.93	15096.26	1.25	19431.98	22549.62	0.86
