# Supplementary material for: Natural variation of TBR confers plant zinc toxicity tolerance through root cell wall pectin methylesterification
Source: Nat Commun. 2024 Jul 11;15:5823. doi: 10.1038/s41467-024-50106-5 (PMC11239920; doi:10.1038/s41467-024-50106-5)
Supplement: Supplementary file 3 — Description of Additional Supplementary Files [file 41467_2024_50106_MOESM3_ESM.pdf]

Description of Additional Supplementary Files:

Supplementary Data 1. 317 accessions used in this study.

Supplementary Data 2. Relative root length (%) in high Zn conditions of 317 natural accessions over 8 days after germination.

Supplementary Data 3. List of 21 significant loci associated with Zn toxicity tolerance.

Supplementary Data 4. Significantly associated SNPs in vicinity of *FRD3*.

Supplementary Data 5. Transcription factor binding motifs in the promoter of *FRD3* gene.

Supplementary Data 6. List of primers used in this study.
