# Supplementary material for: Natural variation of TBR confers plant zinc toxicity tolerance through root cell wall pectin methylesterification
Source: Nat Commun. 2024 Jul 11;15:5823. doi: 10.1038/s41467-024-50106-5 (PMC11239920; doi:10.1038/s41467-024-50106-5)
Supplement: Supplementary file 10 — Reporting Summary [file 41467_2024_50106_MOESM10_ESM.pdf]

nature portfolio

Corresponding author(s): Baohai Li, Peisong Hu & Wolfgang Busch

Last updated by author(s): Jun 16, 2024

Reporting Summary

Nature Portfolio wishes to improve the reproducibility of the work that we publish. This form provides structure for consistency and transparency in reporting. For further information on Nature Portfolio policies, see our Editorial Policies and the Editorial Policy Checklist.

Statistics

For all statistical analyses, confirm that the following items are present in the figure legend, table legend, main text, or Methods section.

n/a	Confirmed	
☐	☒	The exact sample size (*n*) for each experimental group/condition, given as a discrete number and unit of measurement
☐	☒	A statement on whether measurements were taken from distinct samples or whether the same sample was measured repeatedly
☐	☒	The statistical test(s) used AND whether they are one- or two-sided<br>*Only common tests should be described solely by name; describe more complex techniques in the Methods section.*
☒	☐	A description of all covariates tested
☐	☒	A description of any assumptions or corrections, such as tests of normality and adjustment for multiple comparisons
☐	☒	A full description of the statistical parameters including central tendency (e.g. means) or other basic estimates (e.g. regression coefficient) AND variation (e.g. standard deviation) or associated estimates of uncertainty (e.g. confidence intervals)
☐	☒	For null hypothesis testing, the test statistic (e.g. *F*, *t*, *r*) with confidence intervals, effect sizes, degrees of freedom and *P* value noted<br>*Give P values as exact values whenever suitable.*
☒	☐	For Bayesian analysis, information on the choice of priors and Markov chain Monte Carlo settings
☒	☐	For hierarchical and complex designs, identification of the appropriate level for tests and full reporting of outcomes
☒	☐	Estimates of effect sizes (e.g. Cohen's *d*, Pearson's *r*), indicating how they were calculated

Our web collection on statistics for biologists contains articles on many of the points above.

Software and code

Policy information about availability of computer code

Data collection	BRAT (Slovak et al. 2014) was used to collect root length data; Fiji was used to collect root length data and fluorescence signals; CFX Manager Software (Version 1.6, Bio-Rad) was used to collect real-time PCR data.
Data analysis	GWA-portal was used for GWAS analysis; Microsoft Excel 2016 and GraphPad Prism 8.0 were used to analyze the data; IBM SPSS Statistics 24 was used for statistical analysis.

For manuscripts utilizing custom algorithms or software that are central to the research but not yet described in published literature, software must be made available to editors and reviewers. We strongly encourage code deposition in a community repository (e.g. GitHub). See the Nature Portfolio guidelines for submitting code & software for further information.

Data

Policy information about availability of data

All manuscripts must include a data availability statement. This statement should provide the following information, where applicable:

- Accession codes, unique identifiers, or web links for publicly available datasets
- A description of any restrictions on data availability
- For clinical datasets or third party data, please ensure that the statement adheres to our policy

Uncropped gel and blot are provided in Source data. Source data of this paper are provided in the supplementary files.

Research involving human participants, their data, or biological material

Policy information about studies with human participants or human data. See also policy information about sex, gender (identity/presentation), and sexual orientation and race, ethnicity and racism.

Reporting on sex and gender	N.A.
Reporting on race, ethnicity, or other socially relevant groupings	N.A.
Population characteristics	N.A.
Recruitment	N.A.
Ethics oversight	N.A.

Note that full information on the approval of the study protocol must also be provided in the manuscript.

Field-specific reporting

Please select the one below that is the best fit for your research. If you are not sure, read the appropriate sections before making your selection.

[x] Life sciences   [ ] Behavioural & social sciences   [ ] Ecological, evolutionary & environmental sciences

For a reference copy of the document with all sections, see nature.com/documents/nr-reporting-summary-flat.pdf

Life sciences study design

All studies must disclose on these points even when the disclosure is negative.

Sample size	The sample size was determined to be adequate based on the statistical standard in the plant biology.
Data exclusions	No data was excluded.
Replication	All the experiments were repeated at least two times, and similar results were obtained.
Randomization	Plants were grown side-by-side randomly in the growth chamber. The seedlings were selected randomly or selected all for relevant experiments. For liquid treatments, seedlings were distributed randomly in each well of six well plate.
Blinding	Investigators were not blinded to the allocation in the experiments. This because plants can exhibit phenotypes with different treatments that make them visually identifiable. And the phenotype was observed by more than one author.

Reporting for specific materials, systems and methods

We require information from authors about some types of materials, experimental systems and methods used in many studies. Here, indicate whether each material, system or method listed is relevant to your study. If you are not sure if a list item applies to your research, read the appropriate section before selecting a response.

Materials & experimental systems

n/a	Involved in the study	
[ ]	[x]	Antibodies
[x]	[ ]	Eukaryotic cell lines
[x]	[ ]	Palaeontology and archaeology
[x]	[ ]	Animals and other organisms
[x]	[ ]	Clinical data
[x]	[ ]	Dual use research of concern
[ ]	[x]	Plants

Methods

n/a	Involved in the study	
[x]	[ ]	ChIP-seq
[x]	[ ]	Flow cytometry
[x]	[ ]	MRI-based neuroimaging

Antibodies

Antibodies used	Anti-HA-peroxidase, High-Affinity clone (12013819001, Roche); Anti β-Actin clone (CW0264M, CWBIO); Goat Anti-Mouse IgG (H+L)-HRP conjugated (CW0102, CWBIO).
Validation	The antibodies used here have been commonly used for plant science related studies and validated by supplier.

Plants

Seed stocks	The Arabidopsis mutants:frd3-3, frd3-7, tbr-1, tbr-3, at5g06710; The Arabidopsis natural accessions: Eds-1, Eds-9, TRÄ 01, Näs 2, Vår2-6, Sim-1, Fri 1, Spro 1, Bay-0; Lotus japonicus mutant: 30009393.
Novel plant genotypes	For Ostbl31 and Ostbl32 knockout lines, a single guide RNA was designed using the CRISPR-P tool and then transferred into the pBGK032 binary vector. The pBGK032-OsTBL constructs was transformed into the ZH11 rice variety via agrobacterium-mediated transformation. The pTBR::TBR-HA-mCITRINE-HA transgenic lines were generated by the gateway system and then transformed into tbr-3 using the agrobacterium-based floral dipping method.
Authentication	frd3-3, frd3-7, tbr-1, tbr-3, at5g06710, 30009393, Ostbl 31 and Ostbl 32 were verified by genotyping. The allelic transgenic lines and site-directed mutagenesis line were verified based on the glufosinate antibiotic and fluorescence signal.
